# Early Dapagliflozin and Melatonin Treatment Ameliorated LV Fibrosis via Suppressing TGF‐β1/Smads and Activating Nrf2‐ARE Signaling in MI Rodent

**DOI:** 10.1002/kjm2.70205

**Published:** 2026-04-02

**Authors:** Jiunn‐Jye Sheu, Jui‐Ning Yeh, Han‐Tan Chai, John Y. Chiang, Chi‐Ruei Huang, Jun Guo, Hon‐Kan Yip

**Affiliations:** ^1^ Division of Thoracic and Cardiovascular Surgery, Department of Surgery Kaohsiung Chang Gung Memorial Hospital and Chang Gung University College of Medicine Kaohsiung Taiwan, ROC; ^2^ Institute for Translational Research in Biomedicine Kaohsiung Chang Gung Memorial Hospital Kaohsiung Taiwan, ROC; ^3^ Center for Shockwave Medicine and Tissue Engineering Kaohsiung Chang Gung Memorial Hospital Kaohsiung Taiwan, ROC; ^4^ Institute of Nephrology and Blood Purification, the First Affiliated Hospital of Jinan University Jinan University Guangzhou People's Republic of China; ^5^ Department of Cardiology, the First Affiliated Hospital Jinan University Guangzhou People's Republic of China; ^6^ Division of Cardiology, Department of Internal Medicine Kaohsiung Chang Gung Memorial Hospital and Chang Gung University College of Medicine Kaohsiung Taiwan, ROC; ^7^ Department of Computer Science and Engineering National Sun Yat‐Sen University Kaohsiung Taiwan, ROC; ^8^ Department of Healthcare Administration and Medical Informatics Kaohsiung Medical University Kaohsiung Taiwan, ROC; ^9^ Department of Medical Research, China Medical University Hospital China Medical University Taichung Taiwan, ROC; ^10^ School of Medicine, College of Medicine Chang Gung University Taoyuan Taiwan, ROC

**Keywords:** acute myocardial infarction, fibrosis, LV remodeling, Nrf2‐ARE signaling, TGF‐β/Smads signaling

## Abstract

This study tested whether combined dapagliflozin (DAPA) and melatonin (Mel) therapy was superior to merely one for ameliorating the left ventricular (LV) fibrosis/remodeling and improving LV ejection fraction (LVEF) in rats after acute myocardial infarction (AMI). In vitro study demonstrated that DAPA treatment significantly suppressed the TGF‐β/Smads signaling, whereas Mel treatment significantly upregulated Nrf2/ARE signaling in ischemia–reperfusion (IR) H9C2 cells (all *P<* 0.001). Additionally, simultaneously silencing TGF‐β and overexpression of Nrf2 in H9C2 cells significantly suppressed fibrotic and upregulated antioxidant signaling (all *P<* 0.001). Adult male Sprague–Dawley rats were categorized into groups 1 (sham‐operated‐control)/2 (AMI)/3 (AMI + DAPA)/4 (AMI + Mel)/5 (AMI + DAPA‐Mel), and the hearts were harvested by day 28. By day 28 after AMI induction, the LVEF was highest in group 1, lowest in group 2, and significantly higher in group 5 than in groups 3 and 4, but it did not differ between groups 3 and 4, whereas the LV systolic/diastolic dimensions exhibited an opposite pattern of LVEF among the groups (all *P<* 0.0001). The protein expressions of TGF‐β/Smads signaling exhibited an opposite pattern, whereas the protein expressions of Nrf2/ARE signaling displayed an identical pattern of LVEF among the groups (all *P<* 0.0001). The protein expressions of oxidative‐stress/DNA‐mitochondria damaged/inflammatory biomarkers and histopathological/anatomical findings demonstrated that the infarct and fibrotic areas/LV‐chamber dimensions/cardiomyocyte size exhibited an opposite manner of LVEF among the groups (all *P<* 0.0001). In conclusion, combined DAPA‐Mel therapy was superior to merely one for improving LVEF and inhibiting fibrosis/LV remodeling in AMI rodents.

**Abbreviations**
ACEIs: angiotensin converting enzyme inhibitors
AMI: acute myocardial infraction
*ARBs*
: angiotensin II receptor blockers
ARE: antioxidant response element
CRS: cardiorenal syndrome
CVD: cardiovascular disease
DAPA: dapagliflozin
ECM: extracellular matrix
GPx: glutathione peroxidase
HF: heart failure
HO‐1: heme oxygenase
Hsp: heat shock protein
IF: immunofluorescent
IHC: immunohistochemical
IR: ischemia–reperfusion
LV: left ventricular
LVEF: left ventricular ejection fraction
LVEDD: left ventricular end‐diastolic diameter
LVESD: left ventricular end‐systolic diameter
Mel: melatonin
MMP: matrix metalloproteinase
NQO1: NAD(P)H quinone dehydrogenase 1
Nrf2: nuclear factor E2‐related factor 2
PCI: percutaneous coronary intervention
p‐DRP1: phosphorylated dynamin‐related protein 1
p‐NF‐κB: phosphorylated nuclear factor kappa beta
ROS: reactive oxygen species
SGLT2: sodium‐glucose cotransporter 2
SOD1: *superoxide dismutase* 1
TIMP1: tissue inhibitor of metalloproteinase‐1
TNF‐α: tumor necrosis factor alpha
TGF‐β: transforming growth factor beta

## 1 Introduction

Despite state‐of‐the‐art advancement in pharmacomodulations, including thrombolytic therapy [1, 2], angiotensin converting enzyme inhibitors (ACEIs)/angiotensin II receptor blockers (*ARBs*) [3, 4], beta‐blockers [3, 5], and anti‐platelet agents [6, 7], primary percutaneous coronary intervention (PCI) for acute myocardial infarction (AMI) with and without circulatory mechanical support [6, 8, 9, 10] or emergent coronary artery bypass surgery [11, 12] and revised education for primary and secondary prevention [13], over several decades, cardiovascular disease (CVD) is still the first of the top 10 causes of death worldwide. Especially, the AMI remains the leading cause of mortality and morbidity worldwide among hospitalized patients for CVD, regardless of reperfusion therapy [14].

The incidences of heart failure (HF) and poorer long‐term prognostic outcomes in those AMI patients even undergoing reperfusion therapy remain unacceptably high [14, 15]. Studies have further clearly identified that the prognosis of AMI is mainly associated with the development of decompensated HF [16, 17]. These aforementioned issues highlight that the treatment of AMI is an unmet need, which raises an urgency for finding a new therapeutic strategy for those AMI patients, especially for those who are refractory to the conventional treatment.

It is well recognized that myocardial fibrosis in left ventricular (LV) myocardium after AMI is characterized by activation of fibroblasts and accumulation of collagen fibers in the extracellular matrix (ECM) [18, 19] that occupy the space due to lost myocardium after AMI. Additionally, abundant data have shown that myocardial fibrosis is strongly correlated to the LV remodeling [20, 21, 22, 23], which is a critical predictor of HF [22, 23]. Furthermore, myocardial fibrosis is an extremely important prognostic marker in the development of adverse cardiovascular events such as decompensated HF, malignant arrhythmia, and death after AMI [20, 21, 22, 23].

The basic research has proved that AMI invariably triggers an overwhelming inflammatory response characterized by proinflammatory cytokine release, inflammatory cell infiltration, and excessive oxidative stress/reactive oxygen species (ROS) production within the damaged or necrotic myocardium. These pathological processes subsequently activate fibroblasts and promote myocardial fibrosis, which has been recognized as a critical determinant of adverse clinical outcomes following AMI [24, 25, 26]. This raises the need for looking forward to an effective modality for attenuating the LV fibrosis, remodeling, and inflammation, as well as the generation of ROS in patients after AMI.

The nuclear factor erythroid 2‐related factor 2–antioxidant response elements (Nrf2–ARE) signaling pathway, a ROS‐responsive transcriptional system with multiple downstream targets, plays a central role in inducing diverse detoxification enzymes and activating key endogenous antioxidant defenses [27, 28, 29]. Of particularly interesting is that the Nrf2‐AREs signaling has been previously reported as a new therapeutic target for cure of HF [28, 29] and liver cirrhosis [27], suggesting that activation of the Nrf2–ARE‐mediated antioxidant pathway, coupled with inhibition of the profibrotic transforming growth factor (TGF)‐β1/Smad3 signaling cascade in the myocardium, may be critical for interrupting the onset and progression of heart failure, thereby highlighting potential therapeutic targets for mitigating myocardial fibrosis. Our recent study demonstrated that dapagliflozin (DAPA), a sodium–glucose cotransporter‐2 (SGLT2) inhibitor, markedly attenuated renal fibrosis in cardiorenal syndrome (CRS) by suppressing inflammation, apoptosis, fibrosis, and oxidative stress, while concurrently improving endothelial function and ameliorating heart failure [30]. Additionally, our previous studies have demonstrated that melatonin (Mel) exerts potent anti‐inflammatory, immunomodulatory, antioxidative, and antifibrotic effects, as well as anti‐apoptotic actions, primarily through the upregulation of endogenous antioxidant systems and the preservation of mitochondrial functional integrity [31, 32, 33]. Based on these considerations, it was reasonable to hypothesize that combined DAPA therapy—an SGLT2 inhibitor—with Mel may exert additive or potentially synergistic cardioprotective effects, thereby providing superior protection to the LV myocardium in AMI rats by attenuating myocardial fibrosis, limiting adverse LV remodeling and heart failure progression, and enhancing antioxidant capacity.

## 2 Materials and Methods

### 2.1 Ethical Issues

All animal procedures were approved by the Institutional Animal Care and Use Committee (IACUC) of Kaohsiung Chang Gung Memorial Hospital (Affidavit of Approval of Animal Use Protocol No. 2021032202) and were conducted in accordance with the *Guide for the Care and Use of Laboratory Animals*. This study adhered to internationally accepted standards for animal research, including the 3Rs principle, and followed the ARRIVE guidelines to ensure rigorous and ethical reporting of experiments involving live animals.

All animals were housed in an Association for Assessment and Accreditation of Laboratory Animal Care International (AAALAC; Frederick, MD, USA)‐accredited facility at our institution under controlled environmental conditions (temperature: 24°C; light/dark cycle: 12/12 h).

### 2.2 Cell Culture and Procedure and Protocol for Ischemia–Reperfusion (IR) Injury

The procedure and protocol have been described in our recent report [34]. In cell IR model, the H9C2 cells (i.e., 1.5 × 10^6^ cells) were seeded in 10 cm dish. After overnight incubation, the culture medium was replaced with a serum‐free culture medium for 24 h (i.e., in starvation condition) and then incubated in a hypoxic incubator (i.e., 1.0% O_2_ + 5.0% CO_2_) for 3 h. After hypoxia‐condition treatment, the culture medium was exchanged with a complete medium, followed by putting into DAPA treatment or treated with Mel. After additional incubation for 24 h, the cells were collected, and the protein extract was performed with RIPA buffer for western blot. For the immunofluorescent (IF) stain, the same procedure was practiced, but the cells were cultured in EZ Slide (Millicell). After the end of the IR and different procedures treated, the cells were fixed with 4% paraformaldehyde (PFA, Sigma) at room temperature for 15 min. Following the cells being blocked and permeabilized with serum and Triton X‐100, indicated antibodies were used for IF stain. The DAPI staining was applied for counterstaining.

To elucidate the therapeutic impact of DAPA on regulating the TGF‐β/Smad3 signaling, the H9C2 cells were categorized into groups A1 [sham control (SC), i.e., only H9C2 cells (1.6 × 10^6^) were cultured in the dish], A2 [H9C2 cells + ischemia–reperfusion (IR) (i.e., starvation for 24 h and hypoxia for 3 h, then re‐supply the normal culture medium for 48 h)] and A3 [H9C2 cells + IR + DAPA (50 μM for 48 h)].

Additionally, to verify the impact of Mel treatment on augmenting the Nrf2/ARE signaling, the H9C2 cell line was categorized into groups B1 (SC), B2 (H9C2 cells +IR), and B3 [H9C2 cells + IR + Mel (50 μM for 24 h)], respectively. In this study, the dosages of Mel [31, 34] and DPAP [30] were based on our previous studies [30, 31, 34].

### 2.3 Knockdown TGF‐β Gene (i.e., siRNA‐TGF‐β) and Overexpression of Nrf2 Gene in H9C2 Cells

The siRNA TGF‐β1 was purchased from Ambion. Transient transfection of cells with siRNA was performed with GenMute siRNA Transfection Reagent (SignaGen) according to the manufacturer's instructions with slight modifications.

Additionally, for the overexpression of the Nrf2 gene in H9C2 cells, the Nrf2 protein expression vector pcDNA3.1‐Nrf2 was purchased from Genomics Inc. (New Taipei City, Taiwan). Transfection of the cells was conducted with Lipofectamine 3000 according to the manufacturer's instructions, but with slight modifications.

Furthermore, to elucidate whether the suppression of the TGF‐β/Smad3 signaling and enhancement of Nrf2/ARE signaling played the fundamental roles on protecting the cardiomyocytes against IR injury, the H9C2 cells were categorized into groups C1 (SC), C2 (H9C2 cells + IR), and C3 (siRNA‐TGF‐β + overexpression of Nrf2 gene in H9C2 cells + IR), respectively.

### 2.4 Animal Model of AMI Induction and Animal Grouping

Male Sprague–Dawley (SD) rats were utilized in the present study. The procedure and protocol of AMI induction were based on our previous reports [34, 35].

The animals were then categorized into group 1 [Sham‐operated control (SC), that is, only to open the chest wall, followed by closing the muscle layer and skin], group 2 (AMI only, i.e., by ligation of left coronary artery at the level just after first diagonal branch), group 3 [AMI + Mel (50 mg/kg by intraperitoneal injection at 1.5 h, followed by 20 mg/kg/day at days 2 to day 14 after AMI induction)], group 4 [AMI + DAPA (20 mg/kg orally at 1.5 h, followed by 20 mg/kg/day at days 2 to day 14 after AMI induction)] and group 5 (AMI + combined Mel and DAPA), respectively. The dosage of Mel [30] and DAPA [31] were based on our previous reports [30, 35], respectively.

### 2.5 Measurement of LV Ejection Fraction (LVEF) by Using Transthoracic Echocardiography

The procedure and protocol have been described in our previous reports [30, 34, 35]. The 2‐D echo was conducted by an animal cardiologist blinded to the experimental design using an ultrasound machine (Vevo 2100, Visualsonics).

### 2.6 Western Blot Analysis of LV Myocardium

The procedure and protocol for Western blot analysis were based on our recent reports [30, 34, 35]. Briefly, equal amounts (50 μg) of protein extracts were loaded and separated by SDS‐PAGE using acrylamide gradients. The membranes were incubated with the indicated primary antibodies for 1 h at room temperature.

### 2.7 IF Stain for In Vitro Study and Immunohistochemical (IHC) Stain for LV Myocardium

The IF stain for cell culture with antibodies of cytochrome C (1:500) (Santa Cruz), heat shock protein (HSP) 60 (1:1000) (AB 190828). Additionally, the procedure and protocol of IHC stain for heart tissues were based on our previous reports [30, 34, 35].

### 2.8 Statistical Analysis

Quantitative data were expressed as mean ± SD. Statistical analyses were conducted using SAS statistical software for Windows version 8.2 (SAS Institute, Cary, NC, USA). One‐way ANOVA was conducted, followed by Bonferroni multiple comparison post hoc test for comparing variables among groups. A probability value < 0.05 was considered statistically significant.

## 3 Results

### 3.1 Protein Expressions of TGF‐β/Smad3 Signaling Were Markedly Upregulated in IR Condition That Were Effectively Suppressed by DAPA in H9C2 Cells

**FIGURE 1:**
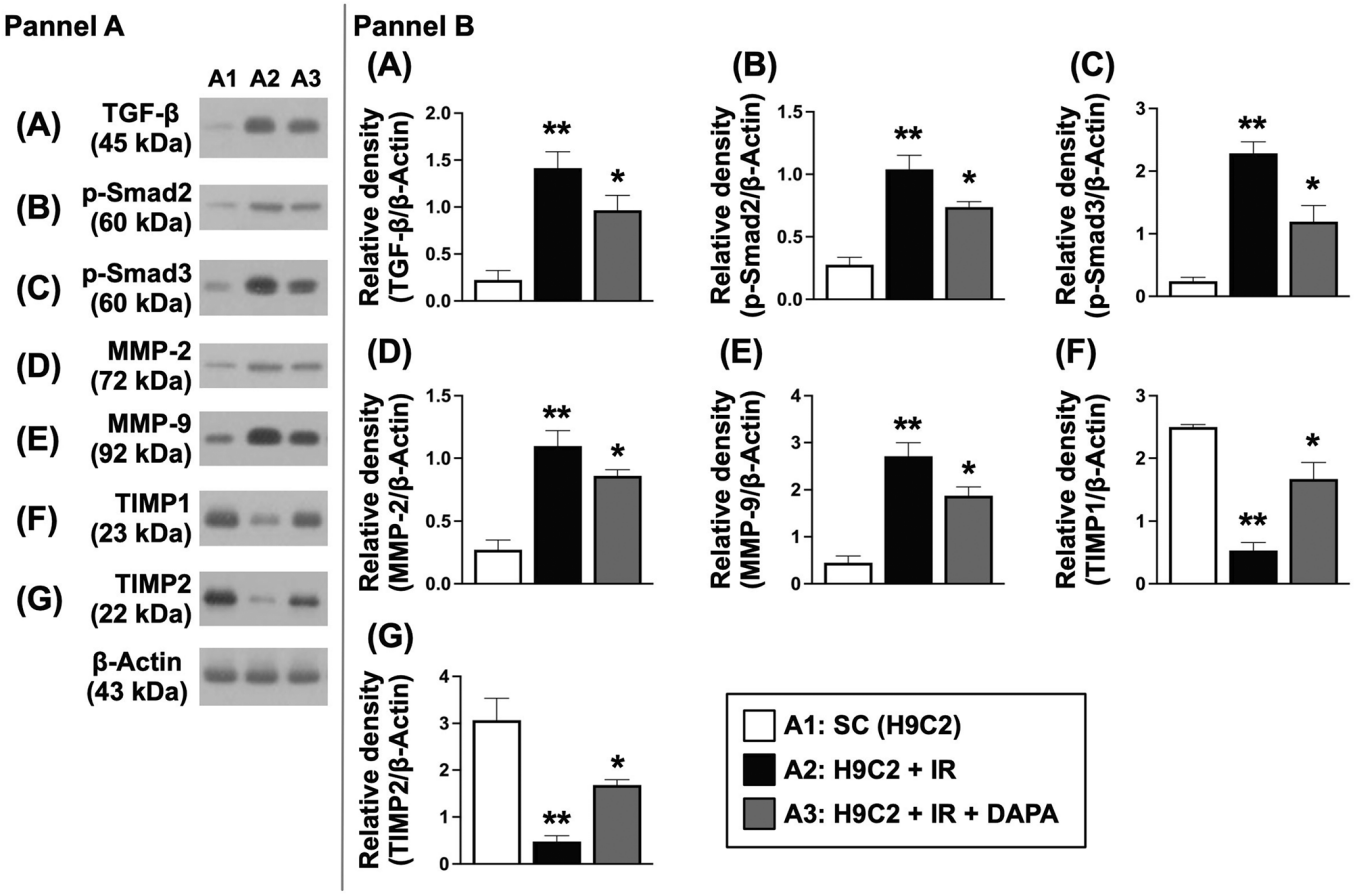
Impact of DAPA on regulating the TGF‐β/Smad3 signaling in IR‐treated H9C2 cells. (A–G) Representative protein expression levels of transforming growth factor (TGF)‐β, phosphorylated Smad2 (p‐Smad2), p‐Smad3, matrix metalloproteinase (MMP)‐2, MMP‐9, Tissue Inhibitor of Metalloproteinases 1 (TIMP1), and TIMP2, respectively. Data are presented as mean ± SD. *P< 0.01, **P< 0.001 versus sham control (SC). Analysis was independently repeated three times (*n* = 3), and consistent trends were observed across experiments. DAPA = dapagliflozin; IR = ischemia–reperfusion. *Note:* Panel (A) displays the representative Western blot bands, whereas panel (B) illustrates the quantitative densitometric analysis presented as a bar graph.

To verify whether DAPA would suppress the IR‐induced activations of upstream and downstream TGF‐β/Smad3 signalings, the Western blot analysis was utilized in the in vitro study (Figure 1). The result showed that the protein expressions of TGF‐β, p‐Smad2, and p‐Smad3, three biomarkers of upstream fibrotic signaling, and protein expressions of matrix metalloproteinase (MMP)‐2 and MMP‐9, two biomarkers of downstream fibrotic signaling, were significantly higher in A2 as compared to A1 that were significantly reversed in A3, whereas the protein expressions of tissue inhibitor of metalloproteinase‐1 (TIMP1) and TIMP2, two antifibrotic indicators, exhibited an opposite pattern of MMPs among the groups.

### 3.2 Protein Expressions of Nrf2/ARE Signaling Were Markedly Suppressed in IR Situation That Were Effectively Upregulated by Mel in H9C2 Cells

**FIGURE 2:**
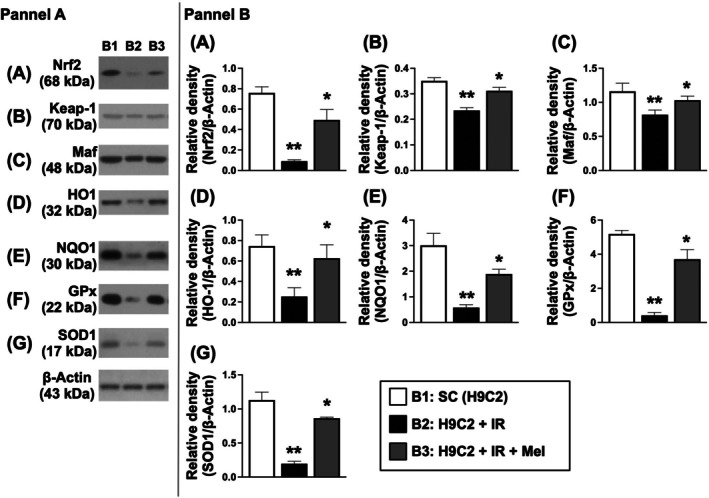
Impact of melatonin (Mel) on Nrf2/ARE signaling in ischemia–reperfusion (IR)–treated H9C2 cells. (A–G) Representative protein expression levels of nuclear factor erythroid 2–related factor 2 (Nrf2), Kelch‐like ECH‐associated protein 1 (Keap‐1), Maf, heme oxygenase‐1 (HO‐1), NAD(P)H quinone dehydrogenase 1 (NQO1), glutathione peroxidase (GPx), and superoxide dismutase 1 (SOD1), respectively. Data are presented as mean ± SD. *P< 0.01, **P< 0.001 versus SC. Analysis was independently repeated three times (*n* = 3), and consistent trends were observed across experiments. Mel = melatonin; IR = ischemia–reperfusion. *Note:* Panel (A) displays the representative Western blot bands, whereas panel (B) illustrates the quantitative densitometric analysis presented as a bar graph.

To elucidate whether Mel treatment could upregulate the Nrf2/ARE signaling against IR induced oxidative stress‐mediated mitochondrial damage, the Western blot analysis was utilized again (Figure 2). The result showed that the protein expressions of Nrf2, keap‐1, and Maf, three biomarkers of upstream Nrf2/ARE signaling, and protein expressions of heme oxygenase 1 (HO‐1), NAD(P)H quinone dehydrogenase 1 (NQO‐1), glutathione peroxidase (GPx), and *superoxide dismutase* 1 (SOD1), four antioxidant biomarkers of downstream Nrf2/ARE signaling, were significantly suppressed in B2 than in B1, that were significantly reversed in B3 (Figure 2).

### 3.3 The Protein Expressions of Fibrotic/Nrf2/ARE Signalings in Situation of Double Gene Manipulations of TGF‐β and Nrf2 in H9C2 Cells Undergoing IR Induction

**FIGURE 3:**
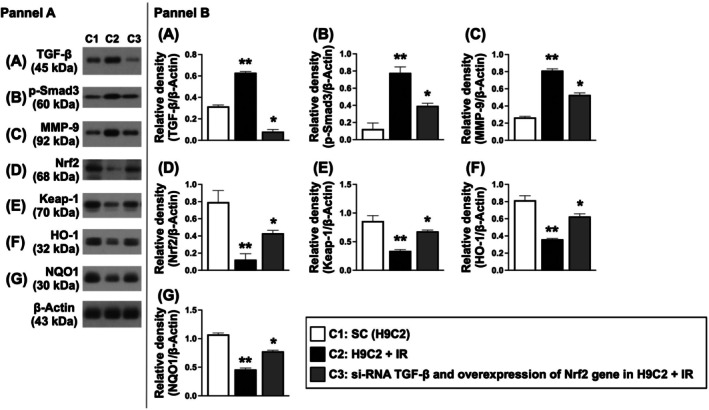
Effects of double gene manipulation of TGF‐β and Nrf2 on fibrotic and Nrf2/ARE‐related protein expressions in ischemia–reperfusion (IR)–treated H9C2 cells. (A–G) Representative protein expression levels of TGF‐β, phosphorylated Smad3 (p‐Smad3), MMP‐9, Nrf2, Keap‐1, HO‐1, and NQO‐1, respectively. Data are presented as mean ± SD. *P< 0.01, **P< 0.001 versus SC. Analysis was independently repeated three times (*n* = 3), and consistent trends were observed across experiments. *Note:* Panel (A) displays the representative Western blot bands, whereas panel (B) illustrates the quantitative densitometric analysis presented as a bar graph.

To verify if the downregulating TGF‐β gene and upregulating Nrf2 genes play the crucial roles on protecting the cardiomyocytes against IR injury, double gene manipulations, that is, through silencing (i.e., knockdown) the TGF‐β gene and overexpression of Nrf2 gene in H9C2 cell line, were conducted in the in vitro study (Figure 3). The result demonstrated that the protein expression of TGF‐β was significantly reduced in C1 (H9C2) and more significantly reduced in C3 (siRNA‐TGF‐β + overexpression of Nrf2 gene in H9C2 cells + IR) than in C2 (H9C2 + IR). Additionally, the protein expressions of TGF‐β, p‐Smad3, and MMP‐9 were significantly lower in C1 than in C2 and C3, and significantly lower in C3 than in C2, whereas the protein expressions of Nrf2, Keap‐1, HO‐1, and NQO‐1 exhibited an opposite manner of p‐Smad3 among the groups. Our findings implicated that the benefits of DAPA and Mel treatments on protecting the cardiomyocytes against IR injury could be mainly through regulating the TGF‐β/Smad2/3 and Nrf2/ARE signaling pathways. Accordingly, based on our in vitro studies, we conducted a preclinical study, that is, by utilizing the combined DAPA and Mel treatment for AMI rodent.

### 3.4 Cellular Levels of Mitochondrial Integrity and DNA‐Damaged Markers in H9C2 Cells Undergoing IR Condition and DAPA‐Mel Treatment

**FIGURE 4:**
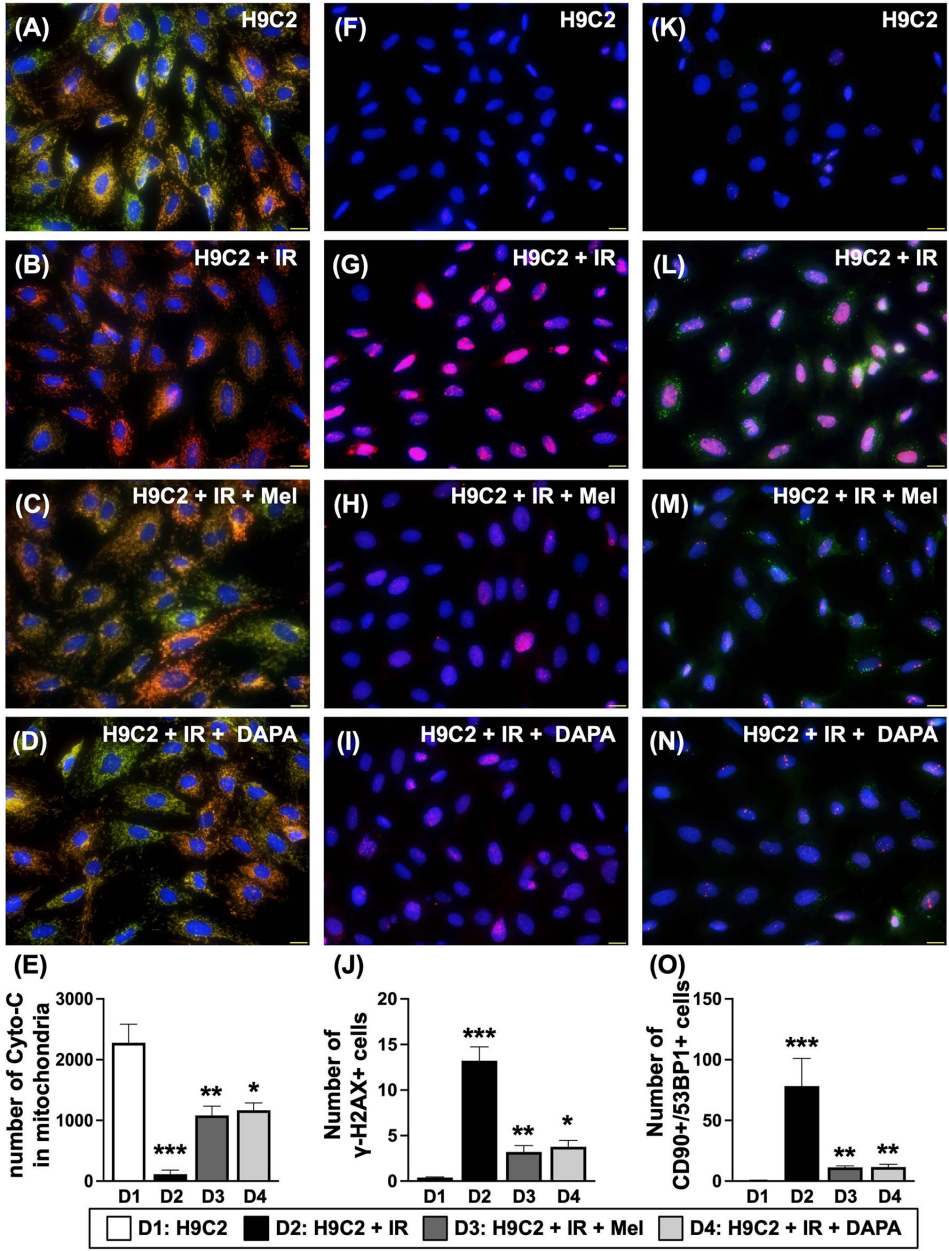
Impact of melatonin (Mel) on mitochondrial cytochrome c retention and DNA damage markers in ischemia–reperfusion (IR)–treated H9C2 cells. (A–D) Representative immunofluorescence (IF) images (400×) showing mitochondrial cytochrome c localization, visualized by double staining for cytochrome c (green) and heat shock protein 60 (HSP60, red); merged images indicate mitochondrial cytochrome c retention. (E) Quantitative analysis of mitochondrial cytochrome c–positive signals. (F–I) Representative IF images (400×) showing γ‐H2AX expression (pink), a marker of DNA double‐strand breaks. (J) Quantitative analysis of γ‐H2AX–positive cells. (K–N) Representative IF images (400×) showing CD90^+^/53BP1^+^ cells, identified by double staining for CD90 (green) and 53BP1 (red); merged images indicate DNA damage–associated cells. (O) Quantitative analysis of CD90^+^/53BP1^+^ cells. Data are presented as mean ± SD. *P< 0.01, **P< 0.001, ***P< 0.0001 versus SC. Nuclei were counterstained with DAPI (blue). Scale bar = 20 μm.

In this in vitro study, the H9C2 cells were categorized into D1 (H9C2), D2 (H9C2 + IR), D3 (H9C2 + IR + Mel), and D4 (H9C2 + IR + DAPA), respectively (Figure 4).

When looked at the cellular level of mitochondria in H9C2, we found that the cellular expression of mitochondrial cytochrome C was significantly lower in D2 than in other groups, significantly lower in D3 and D4 than in D1, but it showed no difference between D3 and D4, implying that Mel or DAPA therapy preserved the integrity of mitochondria in IR‐treated H9C2 cells.

Additionally, the expressions of γ‐H2AX^+^ and CD90^+^/53BP1^+^ cells, two indicators of DNA‐damaged markers, displayed an opposite pattern of mitochondrial cytochrome C, implying that Mel or DAPA therapy attenuated the DNA damage in IR‐treated H9C2 cells.

### 3.5 To Ensure Silencing and Overexpression of Nrf2 Gene Was Effective in Regulating Nrf2/ARE Signaling and Oxidative Stress in H9C2 Cells

**FIGURE 5:**
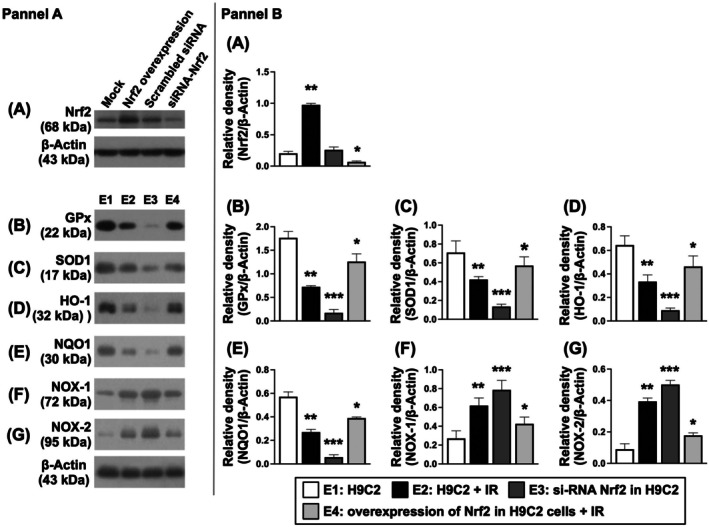
Verification of effective Nrf2 knockdown and overexpression on Nrf2/ARE signaling and oxidative stress in H9C2 cells. (A–G) Representative protein expression levels of GPx, SOD1, HO‐1, NQO1, NOX‐1, and NOX‐2, respectively. Data are presented as mean ± SD. *P< 0.01, **P< 0.001, ***P< 0.0001 versus SC. *Note:* Panel (A) displays the representative Western blot bands, whereas panel (B) illustrates the quantitative densitometric analysis presented as a bar graph.

In this in vitro study, the H9C2 cells were categorized in E1 (H9C2 only), E2 (H9C2 cells + IR), E3 (si‐RNA Nrf2 in H9C2 cells + IR), and E4 (overexpression of Nrf2 in H9C2 cells + IR), respectively (Figure 5). Additionally, the Western blot analysis was applied to verify our hypothesis, and the result demonstrated that the protein expressions of GPX1, SOD1, NQO1, and HO‐1, four indices of downstream biomarkers Nrf2/ARE signaling, were lowest in E3, highest in E1, and significantly higher in E4 than in E2. On the other hand, the protein expressions of NOX‐1 and NOX‐2, two indicators of oxidative stress, were lowest in E1, highest in E3, and significantly lower in E4 than E2. These findings supported the procedure of gene manipulation being successfully conducted and workable regardless of knockdown or overexpression of the Nrf2 gene.

### 3.6 Transthoracic Echocardiographic Study for Measuring the Serial Changes of LVEF in AMI Rodent

**FIGURE 6:**
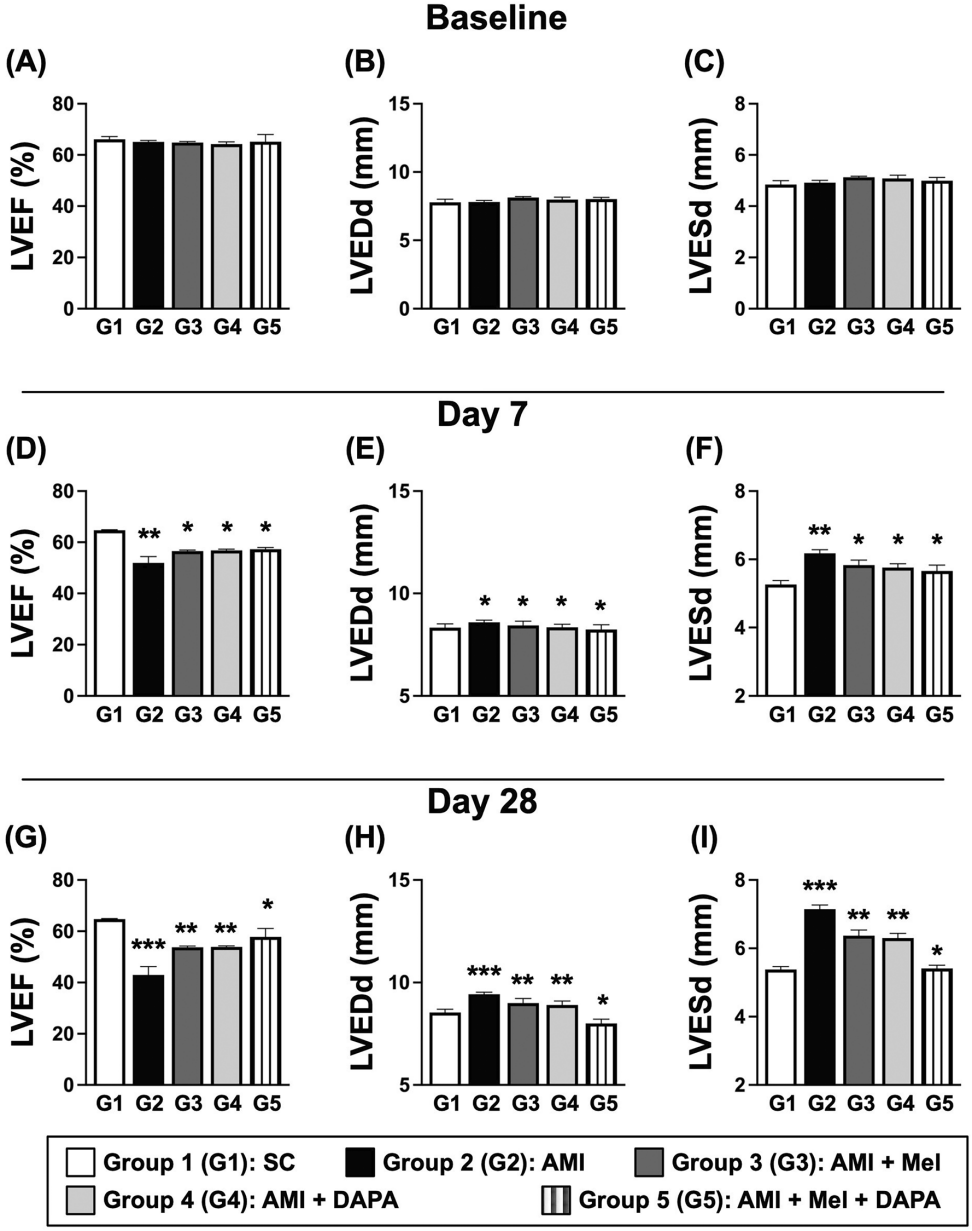
Time‐course transthoracic echocardiographic assessment of left ventricular function in AMI rats. (A–C) Baseline measurements of left ventricular ejection fraction (LVEF), left ventricular end‐diastolic diameter (LVEDd), and left ventricular end‐systolic diameter (LVESd), respectively, showing no significant differences among groups (*P*> 0.5). (D–F) Measurements of LVEF, LVEDd, and LVESd at day 7 post‐AMI.(G–I) Measurements of LVEF, LVEDd, and LVESd at day 28 post‐AMI. Data are presented as mean ± SD (*n* = 8 per group). *P< 0.01, **P< 0.001, ***P< 0.0001 versus SC. Statistical analysis was performed using one‐way ANOVA followed by Bonferroni post hoc test. AMI = acute myocardial infarction.

At the baseline, the LVEF did not differ among the groups. However, by days 7 and 28 after AMI induction, the LVEF was lowest in group 2, highest in group 1, and significantly higher in group 5 than in groups 3 and 4, but it did not show a difference between the two groups at the time points of days 14 and 28 after AMI induction (Figure 6).

Additionally, the LV end‐diastolic diameter (LVEDd) and LV end‐systolic diameter (LVESd) were similar at day 0 prior to AMI induction. However, by day 7 after AMI induction, these two parameters were significantly lower in group 1 than in groups 2–5, but they showed no significant difference among these latter four groups. Moreover, by day 28 after AMI induction, these two parameters were lowest in group 1, highest in group 2, and significantly lower in group 5 than in groups 3 and 4, but they did not differ between groups 3 and 4. Our findings implied that combined DAPA and Mel treatment could be superior to merely one therapy for preserving the LV function and alleviating the LV remodeling in the setting of AMI.

### 3.7 Histopathological and Anatomical Findings of Left Ventricle by Day 28 After AMI Induction

**FIGURE 7:**
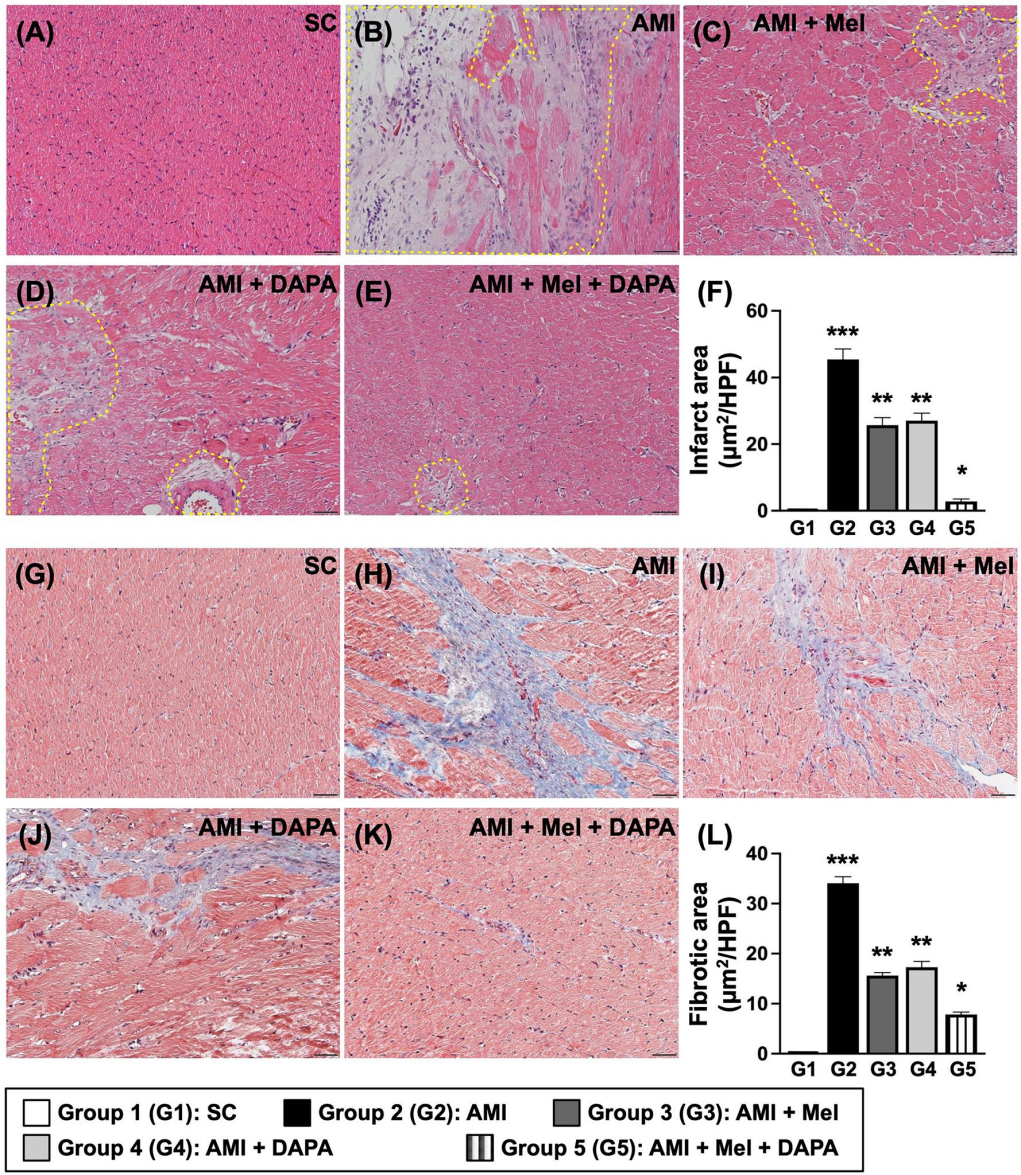
Histopathological assessment of left ventricular remodeling at day 28 after AMI. (A–E) Representative light microscopic images (200×) showing infarcted myocardium (gray area, yellow dotted outline). Scale bar = 50 μm. (F) Quantitative analysis of infarct area. (G–K) Representative Masson's trichrome–stained sections (200×) demonstrating myocardial fibrosis (blue area). Scale bar = 50 μm. (L) Quantitative analysis of fibrotic area. Data are presented as mean ± SD (*n* = 6 per group). *P< 0.01, **P< 0.001, ***P< 0.0001 versus SC. Statistical analysis was performed using one‐way ANOVA followed by Bonferroni post hoc test.

**FIGURE 8:**
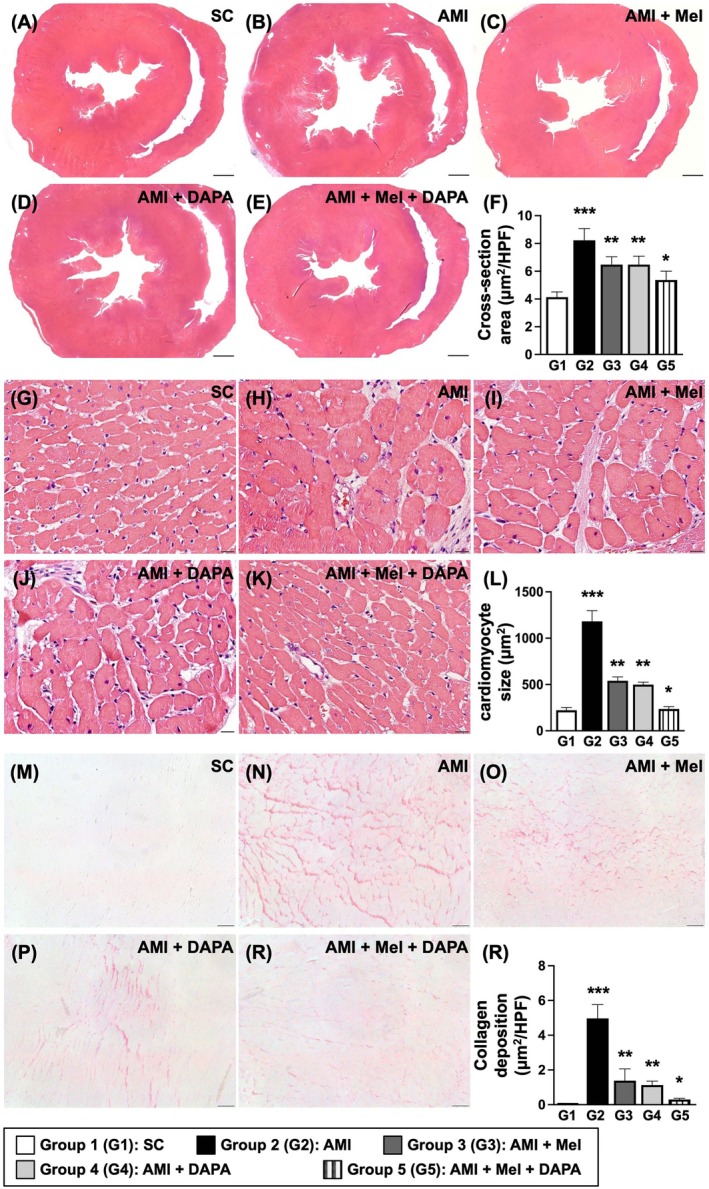
Cross‐sectional area of the mid‐left ventricle, cardiomyocyte size, and collagen deposition at day 28 after AMI induction. (A–E) Representative gross anatomical images of the mid‐ventricular cross‐section used to determine left ventricular cross‐sectional area. (F) Quantitative analysis of left ventricular cross‐sectional area. (G–K) Representative light microscopic images (400×) showing cardiomyocyte size in left ventricular myocardium. (L) Quantitative analysis of cardiomyocyte size. (M–Q) Representative microscopic images (400×) demonstrating collagen deposition (pink). (R) Quantitative analysis of collagen deposition area. Data are presented as mean ± SD. *P< 0.01, **P< 0.001, ***P< 0.0001 versus SC. Statistical analysis was performed using one‐way ANOVA followed by Bonferroni post hoc test (*n* = 6 per group). Scale bar = 20 μm.

Light microscopic findings of the infarct area and Masson's trichrome stain for the fibrotic area in LV myocardium were highest in group 2, lowest in group 1, and significantly lower in group 5 than in groups 3 and 4, but they were similar between these two groups (Figure 7). Additionally, the Sirius red stain demonstrated that the collagen deposition area exhibited a similar pattern of fibrosis among the groups (Figure 8). Furthermore, the anatomical feature of the mid‐cross‐section of the LV‐chamber area and the size of cardiomyocytes exhibited a similar manner of fibrosis among the groups (Figure 8). These findings once again supported that combined DAPA and Mel treatment was better than just one treatment for protecting the LV myocardium and inhibiting the cardiomyocytes/LV‐chamber remodeling.

### 3.8 Protein Expressions of TGF‐β/Smad3 Signaling in LV Myocardium by Day 28 After AMI Induction

**FIGURE 9:**
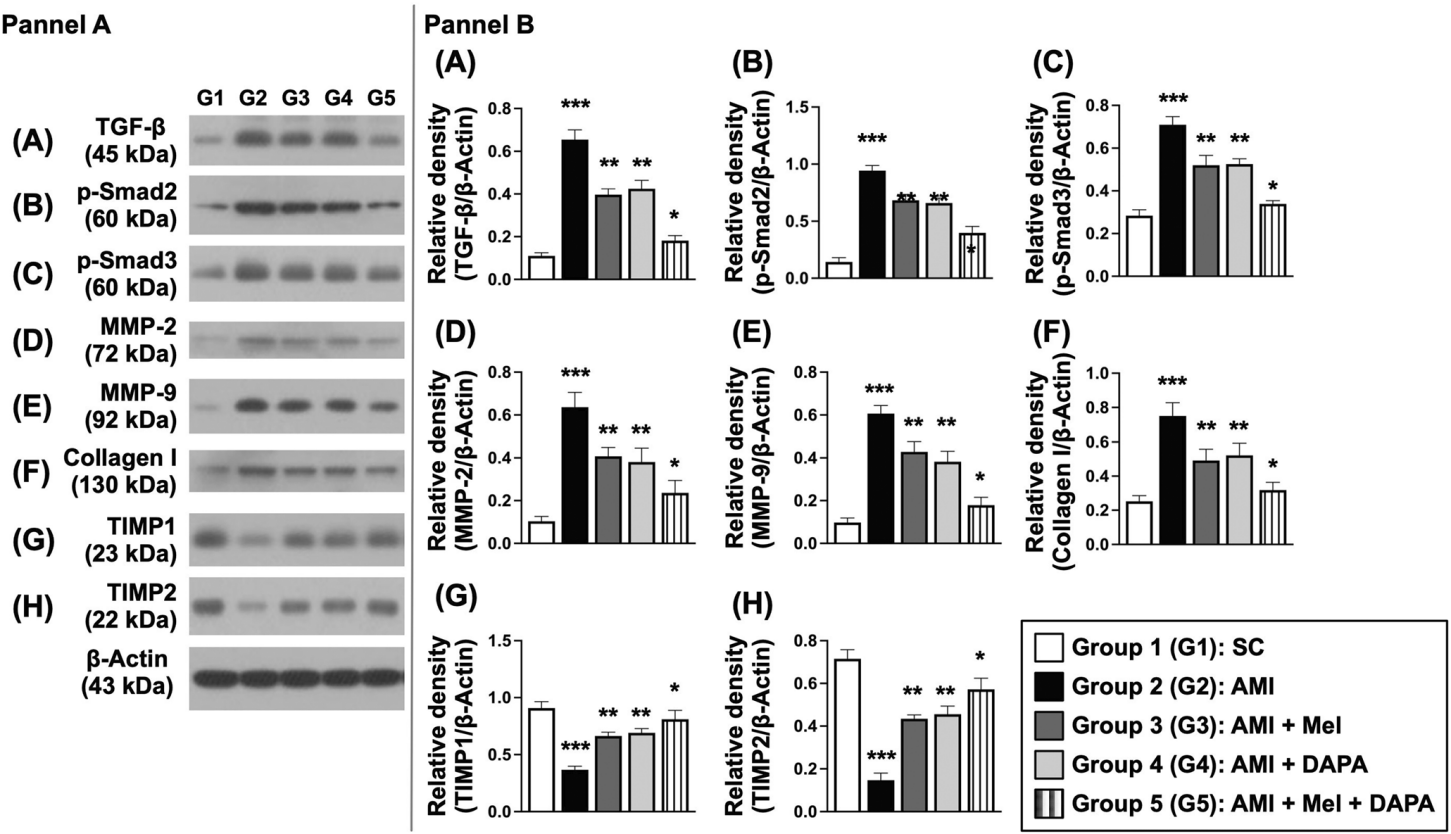
Protein expressions of TGF‐β/Smad3 signaling–related molecules in left ventricular myocardium at day 28 after AMI induction. (A–H) Representative protein expression levels of TGF‐β, p‐Smad3, MMP‐2, MMP‐9, collagen I, TIMP1, and TIMP2, respectively. Data are presented as mean ± SD. *P< 0.01, **P< 0.001, ***P< 0.0001 versus SC. Statistical analysis was performed using one‐way ANOVA followed by Bonferroni post hoc test (*n* = 6 per group). *Note:* Panel (A) displays the representative Western blot bands, whereas panel (B) illustrates the quantitative densitometric analysis presented as a bar graph.

To verify whether the TGF‐β/Smad3 signaling, that is, a fibrosis signaling, would be attenuated by DAPA‐Mel treatment, the Western blot was utilized in the present study (Figure 9). The result showed that the protein expressions of TGF‐β, p‐Smad2, and p‐Smad3, three biomarkers of upstream fibrotic signaling, and the protein expressions of MMP‐2, MMP‐3, collagen I, three indicators of downstream fibrotic signaling, were highest in group 2, lowest in group 1 and significantly higher in groups 3 and 4 than in group 5, but they did not differ between the groups 3 and 4, whereas the protein expressions of TIMP1 and TIMP2, two indicators of antifibrotic markers, exhibited an opposite pattern of MMP‐9 among the groups. Our in vivo findings implied that DAPA‐Mel treatment effectively suppressed fibrotic signaling.

### 3.9 Protein Expressions of Nrf2/ARE Signaling in LV Myocardium by Day 28 After AMI Induction

**FIGURE 10:**
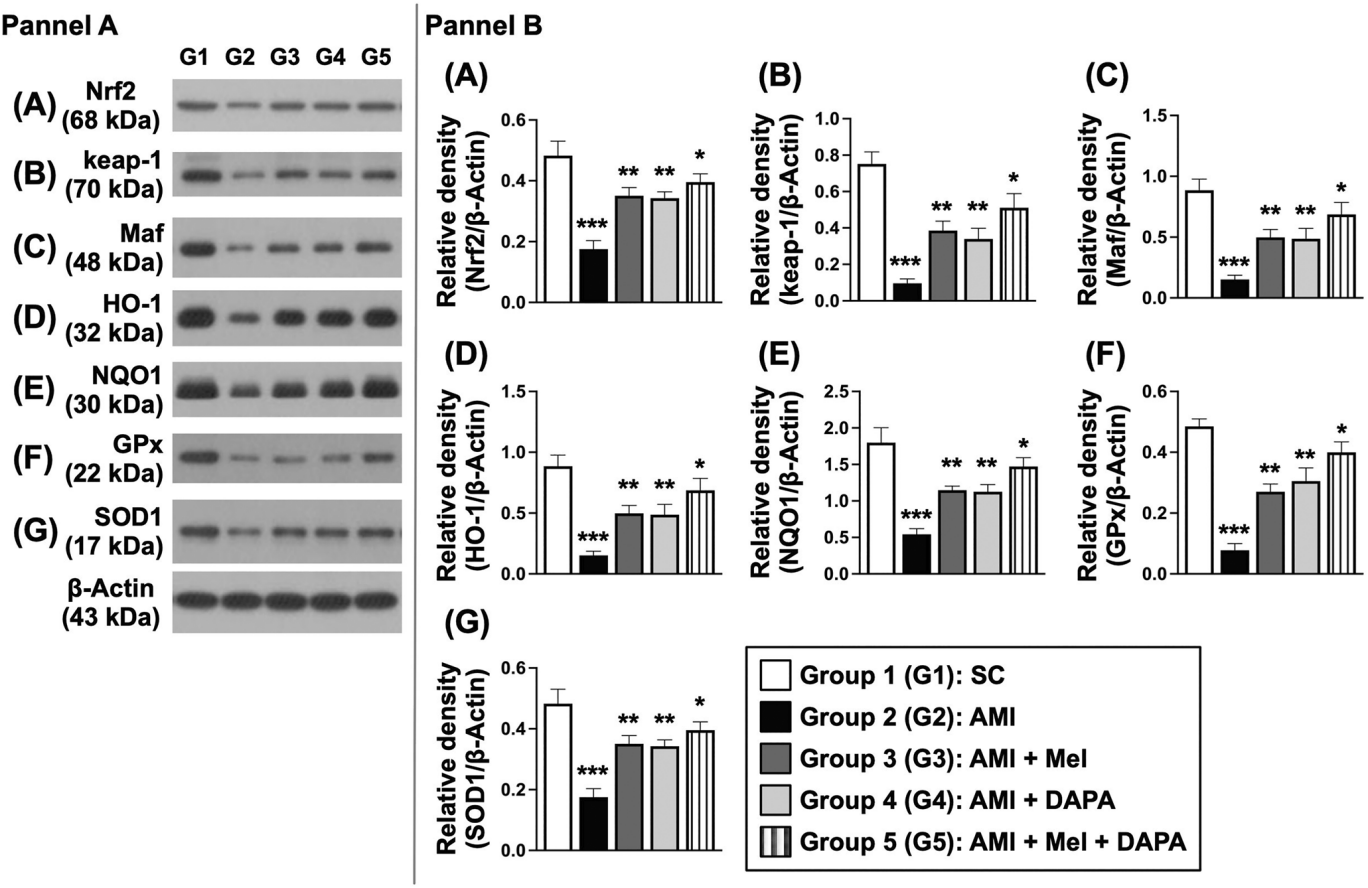
Protein expression levels of Nrf2/ARE signaling–related molecules in left ventricular myocardium at day 28 after AMI induction. (A–G) Representative protein expression levels of Nrf2, Keap‐1, Maf, HO‐1, NQO1, GPx and SOD1, respectively. Data are presented as mean ± SD. *P< 0.01, **P< 0.001, ***P< 0.0001 versus SC. Statistical analysis was performed using one‐way ANOVA followed by Bonferroni post hoc test (*n* = 6 per group). *Note:* Panel (A) displays the representative Western blot bands, whereas panel (B) illustrates the quantitative densitometric analysis presented as a bar graph.

To verify whether the Nrf2/ARE signaling, that is, an antioxidant signaling, would be upregulated by DAPA‐Mel treatment, the Western blot was conducted in the in vivo study (Figure 10). The result showed that the protein expressions of Nrf2, Keap‐1, and Maf, three biomarkers of upstream Nrf2/ARE signaling, and protein expressions of HO‐1, NQO‐1, GPx, and SOD1, four indicators of downstream Nrf2/ARE signaling, were highest in group 5, lowest in group 1, and significantly higher in groups 3 and 4 than in group 1, but they did not differ between groups 3 and 4. Our findings implicated that DAPA‐Mel treatment effectively enhanced the antioxidant in LV myocardium, resulting in protecting the myocardium from oxidative stress damage.

### 3.10 Protein Expressions of Oxidative Stress and Inflammation in LV Myocardium by Day 28 After AMI Induction

**FIGURE 11:**
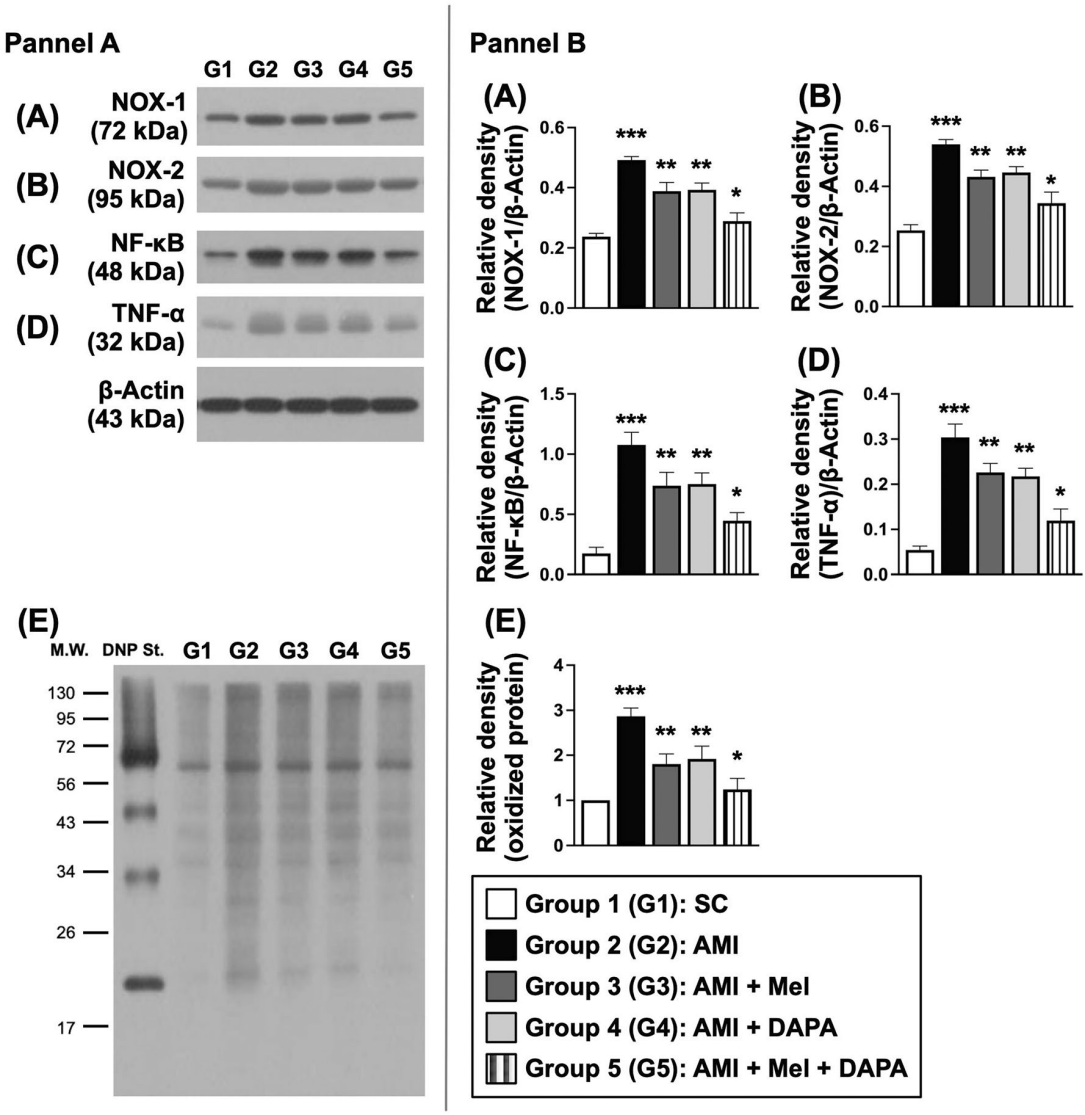
Oxidative stress‐ and inflammation‐related protein expressions in left ventricular myocardium at day 28 after AMI induction. (A–D) Representative protein expression levels of NOX‐1, NOX‐2, NF‐κB, tumor necrosis factor‐α (TNF‐α), and oxidized proteins, respectively. For panel (A), (E) indicated the OxyBlot assay was conducted for the detection of oxidized protein; the left and right lanes represent the molecular weight marker and the control oxidized protein standard, respectively. M.W. = molecular weight; DNP = 1–3‐dinitrophenylhydrazone. Data are presented as mean ± SD. *P< 0.01, **P< 0.001, ***P< 0.0001 versus SC. Statistical analysis was performed using one‐way ANOVA followed by Bonferroni post hoc test (*n* = 6 per group). *Note:* Panel (A) displays the representative Western blot bands, whereas panel (B) illustrates the quantitative densitometric analysis presented as a bar graph.

To assess whether DAPA‐Mel treatment would suppress AMI‐induced oxidative stress and inflammation, the Western blot analysis was performed (Figure 11). The result showed that the protein expressions of NOX‐1, NOX‐2, and oxidized protein, three indicators of oxidative stress, and protein expressions of NF‐κB and TNF‐α, two indicators of inflammation, were lowest in group 1, highest in group 2, and significantly lower in group 5 than in groups 3 and 4, but they demonstrated no difference in the latter two groups.

### 3.11 Protein Expression of Apoptosis, Mitochondrial/DNA‐Damage in LV Myocardium by Day 28 After AMI Induction

**FIGURE 12:**
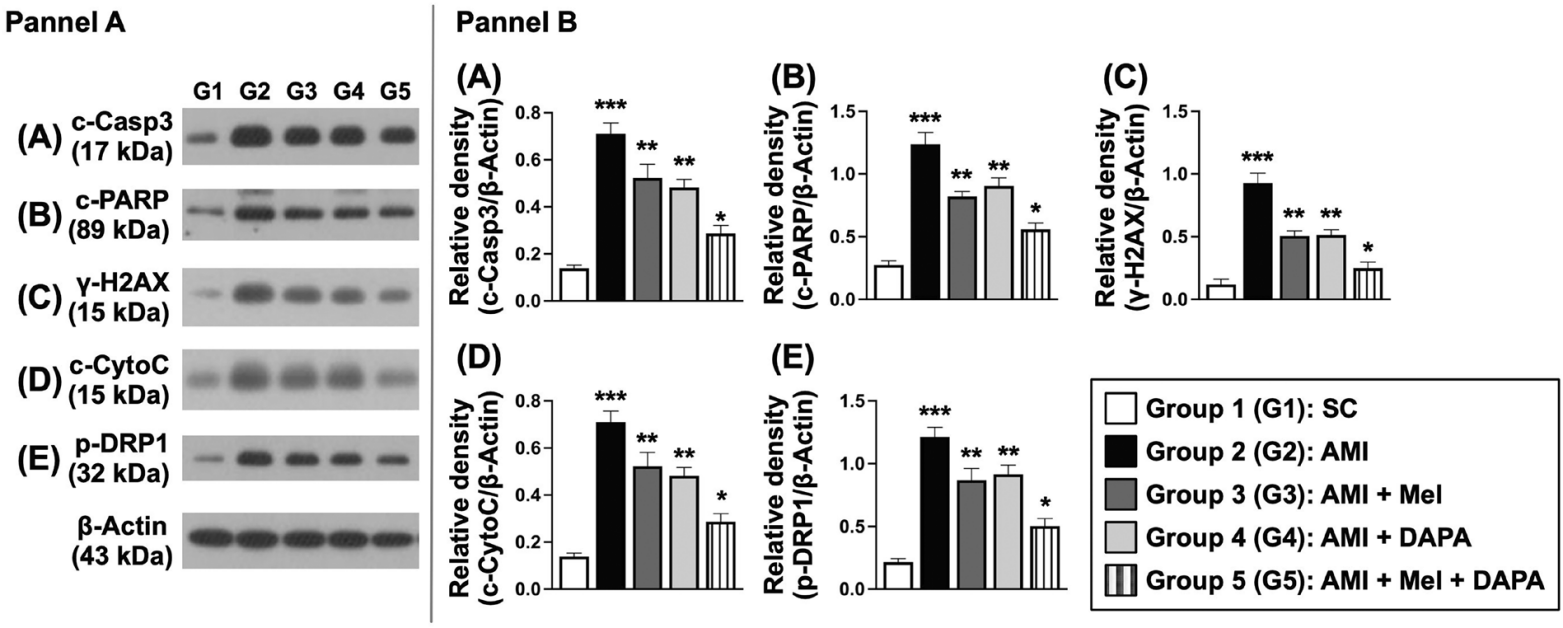
Protein expressions of apoptosis‐ and mitochondrial/DNA damage–related biomarkers in left ventricular myocardium at day 28 after AMI induction. (A–E) Representative protein expression levels of cleaved caspase‐3 (c‐Casp3), cleaved poly (ADP‐ribose) polymerase (c‐PARP), γ‐H2AX, cytosolic cytochrome C (c‐CytoC), and phosphorylated dynamin‐related protein‐1 (p‐DRP1), respectively. Data are presented as mean ± SD. *P< 0.01, **P< 0.001, ***P< 0.0001 versus SC, as determined by one‐way ANOVA followed by Bonferroni post hoc test (*n* = 6 per group). *Note:* Panel (A) displays the representative Western blot bands, whereas panel (B) illustrates the quantitative densitometric analysis presented as a bar graph.

To verify whether the molecular level of myocardial damage markers would be suppressed by DAPA‐Mel treatment, Western blot was once again applied in the present study (Figure 12). As we expected, the protein expressions of cleaved caspase 3 and cleaved PARP, two indices of apoptosis, protein expression of γ‐H2AX, an indicator of DNA damage, and protein expressions of cytosolic cytochrome C and p‐DRP1, two indicators of mitochondrial damage, were lowest in group 1, highest in group 2, and significantly lower in group 5 than in groups 3 and 4, but they were similar between groups 3 and 4, implicating that DAPA‐Mel treatment fruitfully preserved LV myocardium away from AMI‐induced damage.

### 3.12 Safety Concern of High‐Dose DAPA Utilized in the in Vitro Study

To verify that the high dose of DAPA (50 μM) used in the in vitro experiments was safe and non‐toxic to cultured cells, stepwise concentrations of DAPA (0, 5, 12.5, 20, and 50 μM) were administered in the cell‐culture system (Figure S1). Both the MTT viability assay and flow‐cytometric analysis of cellular apoptosis showed no significant differences across these concentrations. These findings indicate that DAPA up to 50 μM did not induce cytotoxicity or an increased apoptosis in the treated cells.

## 4 Discussion

This study, which investigated the impact of DAPA‐Mel therapy on preserving heart function and attenuating the myocardial fibrosis and LV remodeling, had begotten several preclinical striking implications. First, activated TGF‐β signaling and inactivated Nrf2/ARE signaling, which were observed in the setting of AMI, played crucial roles on LV myocardial fibrosis and LV remodeling, resulting in LV functional deterioration. Second, combined DAPA‐Mel treatment inhibits the LV myocardial fibrosis and remodeling and preserves heart function mainly through suppressing the TGF‐β/Smads signaling and activating the Nrf2/ARE signaling, highlighting that this combination regimen would have a great therapeutic potential for patients after AMI.

Generation of fibrosis and accumulation of ECM are two common pathological findings after cardiomyocyte death following AMI [18, 19, 36, 37], resulting in LV remodeling and pump failure and unfavorable clinical outcomes. Thus, to impede the TGF‐β/Smads signaling pathway, which initiates and propagates the tissue/organ fibrosis [24, 25, 26, 38] could be an innovative therapeutic novelty. An essential in vitro finding was that IR markedly augmented upstream and downstream TGF‐β/Smads signaling in H9C2 cells, resulting in the upregulation of the fibrosis biomarkers. Accordingly, our findings corroborated with the findings of the previous studies [18, 19, 24, 25, 26, 36, 37]. Of importance was that DAPA therapy remarkably suppressed the expressions of the fibrotic biomarkers.

It is well recognized that Nrf2/ARE signaling is an extremely important endogenous antioxidative system [27, 28, 29, 39] that has been extensively investigated as a new therapeutic weapon for HF and improvement of heart function [28, 29]. A principal finding in another in vitro study was that the upstream and downstream signaling pathways of Nrf2/ARE were markedly suppressed in H9C2 cells in the situation of IR (i.e., a scenario of myocardial ischemia in the clinical setting of AMI). Of particular importance was that Mel therapy upregulated the expressions of upstream and downstream signaling pathways of Nrf2/ARE in IR H9C2 cells as well as the intracellular mitochondria, which is well known as the energy donor for the cellular metabolic need and survival. In this way, our findings were comparable with the findings from the previous studies [27, 28, 29, 39].

**FIGURE 13:**
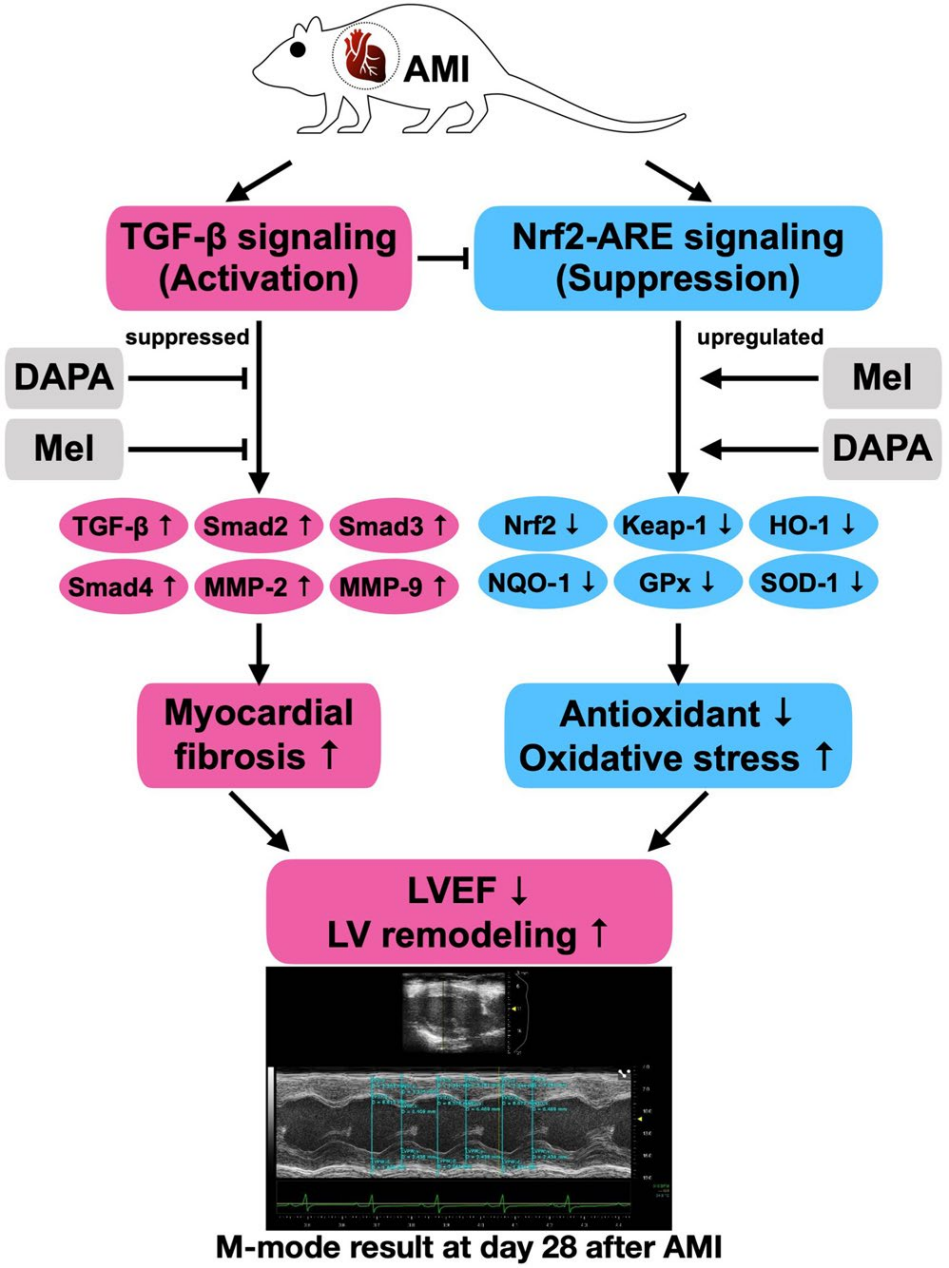
Schematic illustration of the fundamental roles of TGF‐β1/Smad and Nrf2–ARE signaling pathways in regulating cardiac function following AMI. Activation of the TGF‐β1/Smad pathway contributes to myocardial fibrosis, adverse LV remodeling, and deterioration of LVEF, whereas activation of the Nrf2–ARE pathway enhances antioxidant defenses, attenuates oxidative stress, and preserves myocardial structure and function. DAPA and Mel are depicted as modulators that suppress TGF‐β1/Smad signaling while concurrently enhancing Nrf2–ARE signaling, thereby synergistically improving post‐AMI cardiac outcomes.

Meanwhile, although the comprehensive findings of the present study are both attractive and promising, elucidating the integrated mechanisms underlying the combined therapy would further enhance readers' understanding of how these two regimens improve outcomes after AMI. To more precisely delineate the mechanistic basis, we performed a dual gene manipulation experiment in H9C2 cells by simultaneously silencing TGF‐β (siRNA–TGF‐β) and overexpressing Nrf2, herein referred to as double gene–manipulated H9C2 cells (H9C2^DGM^). Intriguingly, the protein expression levels of the TGF‐β/Smad signaling pathway were markedly more suppressed in the H9C2^DGM^ + IR group than in the H9C2 + IR group. Conversely, Nrf2/ARE pathway activation was substantially higher in the H9C2^DGM^ + IR group compared with the H9C2 + IR group. Furthermore, pathway interaction analyses (Figure 13) indicated that modulation of both Nrf2/ARE and TGF‐β/Smad signaling was not solely attributable to either DAPA or melatonin individually; rather, both agents intrinsically possess the capacity to influence these pathways. Collectively, these findings support the mechanistic rationale for early administration of combined DAPA–Mel therapy to prevent myocardial fibrosis and LV remodeling, thereby attenuating LV dysfunction through coordinated suppression of TGF‐β/Smad signaling and enhancement of Nrf2/ARE–mediated antioxidative defense.

Several attractive and promising results were obtained from this preclinical study. First, the impressive finding was that the LVEF was remarkably preserved in groups 3 and 4 and further remarkably preserved in group 5 than in group 2, whereas the LVE remodeling displayed an opposite manner of LVEF among the groups, highlighting that combined DAPA‐Mel therapy should be strongly recommended for those AMI patients. Second, the promising finding was that when we looked at the histopathological findings, we identified that the infarct/fibrosis areas, LV chamber dimension, and the cardiomyocyte size were exceedingly increased in group 2 than were brilliantly ameliorated in groups 3 and 4, and further brilliantly ameliorated in group 5, explaining why the heart function was notably improved and LV remodeling was significantly attenuated in the AMI rodent after receiving DAPA‐Mel treatment.

It is well recognized that inflammation and oxidative stress are significantly associated with the unfavorable outcomes in ischemia‐related organ dysfunction [30, 31, 32, 33, 34, 35]. A cardinal finding was that the inflammatory and oxidative‐stress biomarkers were significantly increased in the AMI group without treatment than in those of AMI groups that received DAPA‐Mel treatment. Our findings, in addition to being consistent with the findings of previous studies [30, 31, 32, 33, 34, 35], once again explained why DAPA‐Mel treatment yielded a better outcome in AMI rodents.

### 4.1 Study Limitations

Several limitations of the present study should be acknowledged. First, despite the comprehensive experimental design, the molecular mechanisms underlying the observed cardioprotective effects are likely more complex than those depicted in Figure 13, which provides a schematic summary derived from our experimental findings. Additional interacting pathways and regulatory networks may contribute to myocardial remodeling following acute myocardial infarction and were not fully explored in the current investigation. Furthermore, circulating biochemical markers of myocardial injury, such as cardiac troponin I or creatine kinase–MB, were not measured, which may limit direct biochemical correlation with myocardial injury severity. Second, the DAPA regimens employed in both in vivo and in vitro experiments represent pharmacological/mechanistic dosing, deliberately selected to amplify intracellular signaling responses and to facilitate mechanistic interrogation of the TGF‐β/Smad and Nrf2/ARE pathways under ischemic and oxidative stress conditions. These exposure levels exceed clinically achievable plasma concentrations and were not intended to model clinical dosing, pharmacokinetics, or predict therapeutic efficacy at standard human doses. Accordingly, the mechanistic insights derived from this study should be interpreted as proof‐of‐concept evidence supporting pathway involvement rather than as direct surrogates for clinical treatment effects.

In conclusion, the results of the present study clarified that TGF‐β/Smads signaling was activated and Nrf2/ARE signaling was suppressed in ischemic cardiomyocytes/AMI. DAPA‐Mel treatment improved LVEF and suppressed LV remodeling mainly through manipulating these two signalings.

## Funding

This work was supported by Chang Gung Memorial Hospital (CMRPG8L1081).

## Ethics Statement

All animal procedures were approved by the Institutional Animal Care and Use Committee of our hospital (Affidavit of Approval of Animal Use Protocol No. 2021032202) and were conducted in accordance with the *Guide for the Care and Use of Laboratory Animals*, 8th edition (NIH Publication No. 85–23, National Academy Press, Washington, DC, USA; revised 2011).

## Conflicts of Interest

The authors declare no conflicts of interest.

## Supporting information


**Figure S1:** Safety concern of high‐dose DAPA utilized in the in vitro study A1 to A3) Illustrating the cell viability at 24, 48, and 72 h under stepwise concentrations of DAPA (0, 5, 12.5, 20, and 50 μM). No statistically significant differences in cell viability were observed among the various DAPA treatment concentrations. (B1 to B4) Illustrating the flow cytometric analysis for identification of Early (annexin V+/PI‐) the late (annexin V+/PI+)phase of apoptosis. No statistically significant differences in cell apoptosis were observed among the various DAPA treatment concentrations.

## Data Availability

The data that support the findings of this study are available from the corresponding author upon reasonable request.
