# Supplementary figures and images for: Early Dapagliflozin and Melatonin Treatment Ameliorated LV Fibrosis via Suppressing TGF‐β1/Smads and Activating Nrf2‐ARE Signaling in MI Rodent
Source: Kaohsiung J Med Sci. 2026 Apr 2:e70205. Online ahead of print. doi: 10.1002/kjm2.70205 (PMC13399667; doi:10.1002/kjm2.70205)

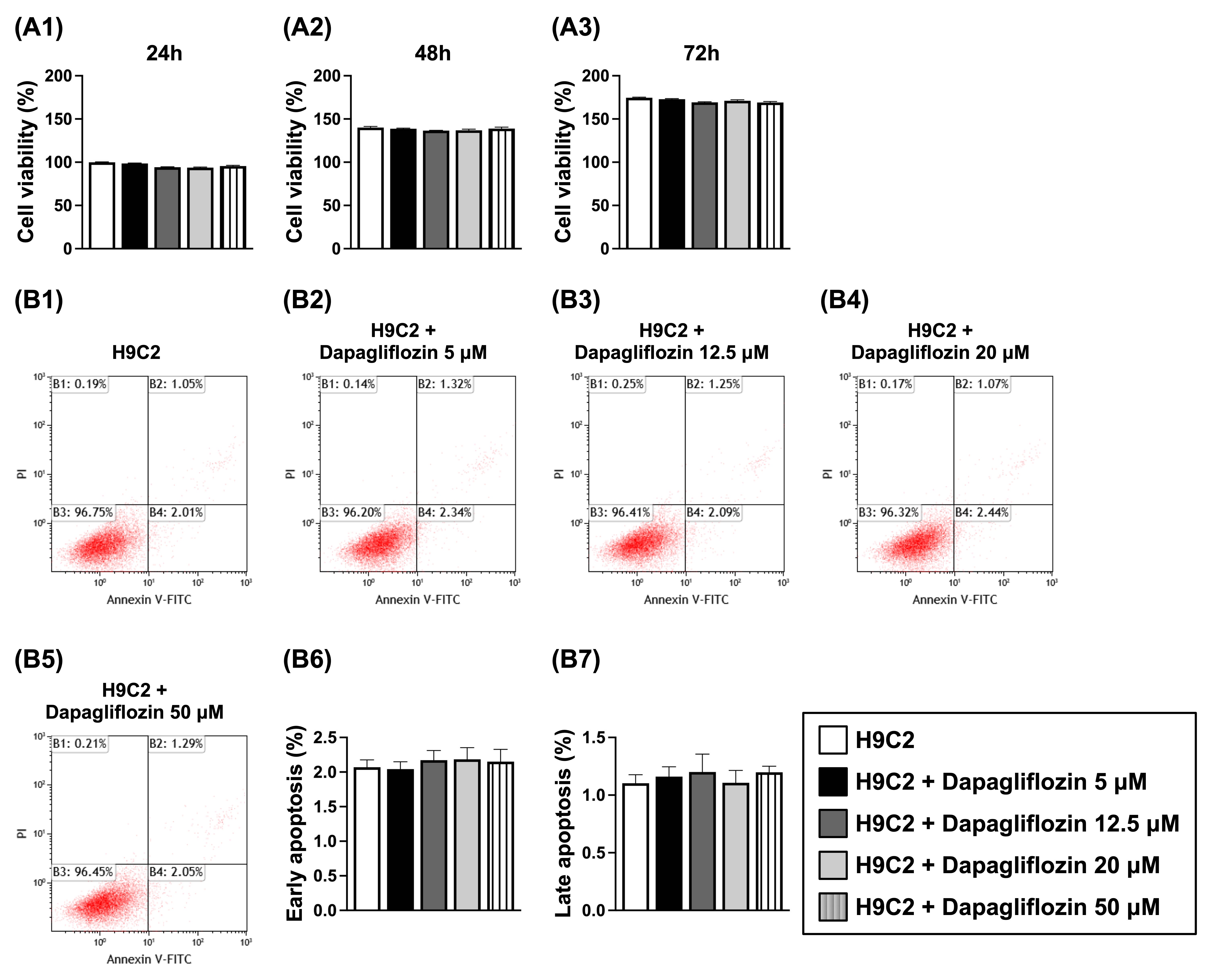

Supplement: Supplementary file 1 — Figure S1: Safety concern of high‐dose DAPA utilized in the in vitro study A1 to A3) Illustrating the cell viability at 24, 48, and 72 h under stepwise concentrations of DAPA (0, 5, 12.5, 20, and 50 μM). No statistically significant differences in cell viability were observed among the various DAPA treatment concentrations. (B1 to B4) Illustrating the flow cytometric analysis for identification of Early (annexin V+/PI‐) the late (annexin V+/PI+)phase of apoptosis. No statistically significant differences in cell apoptosis were observed among the various DAPA treatment concentrations. [file KJM2-9999-e70205-s001.jpg]
